# The Reliability of the Greulich and Pyle Atlas When Applied to a Modern Scottish Population^*^

**DOI:** 10.1111/j.1556-4029.2012.02294.x

**Published:** 2012-10-12

**Authors:** Lucina Hackman, Sue Black

**Affiliations:** 1 Centre for Anatomy and Human Identification, University of DundeeDundee, U.K

**Keywords:** forensic science, forensic anthropology, age estimation, radiographs, Greulich and Pyle atlas, age assessment living

## Abstract

This study examines the reliability of age estimation utilizing the Greulich and Pyle atlas in relation to a modern Scottish population. A total of 406 left-hand/wrist radiographs (157 females and 249 males) were age-assessed using the Greulich and Pyle atlas. Analysis showed that there was a strong correlation between chronological age and estimated age (females *R*^2^ = 0.939, males *R*^2^ = 0.940). When age groups were broken down into year cohorts, the atlas over-aged females from birth until 13 years of age. The pattern for males showed that the atlas under-estimated age until 13 years of age after which point it consistently over-aged boys between 13 and 17 years of age. This study showed that the Greulich and Pyle atlas can be applied to a modern population but would recommend that any analysis takes into account the potential for over- and under-aging shown in this study.

The radiograph of the left hand/wrist is one of the triumvirate of images recommended by the German Study Group on Forensic Age Diagnostics of the German Association of Forensic Medicine (AGFAD) for age estimation in the living (1). Maturity in this skeletal area is estimated using one of a selection of atlases (2–12). One of the most commonly utilized of these is the Greulich and Pyle hand/wrist atlas (5). This study aims to examine the applicability of the Greulich and Pyle atlas to a modern Scottish sample to assess whether it is appropriate for forensic use as a method of age estimation when applied to a contemporary population.

The need for age estimation in the living has escalated in recent decades. The age estimation of those who are unable or unwilling to prove their age, or who may not know their age, has become increasingly common, especially at geographical borders where there is regular movement of people (13,14). Documents can be lost, falsified, or simply may not exist, and as people move across borders or are victims of traffickers, the ability to prove that they have reached defined chronological milestones becomes important to authorities. As the number of disputed age cases has risen, forensic science and its practitioners are also coming under scrutiny in the United Kingdom (15). While the effects of this scrutiny are still embryonic, it has resulted in the questioning of the reliability of conclusions drawn by the application of forensic methodologies. This in turn creates a pressing need for those same methodologies to be revisited and re-examined to evaluate their legal admissibility. The reassessment of reliability and accuracy is an even greater priority when methods are being applied in ways for which they were never designed, as is the case with the Greulich and Pyle atlas, which was originally designed to enable clinical practitioners to assess the development of children as they progress to maturity.

Age estimation in the living depends upon the comparison of radiographic images to reference images. These reference images are collected and collated from a sample of individuals of known sex, age, and background, thus allowing the assessor to gauge the degree of skeletal and dental maturation and then relate this to a chronological age. The left hand/wrist is one of the areas of the body that is commonly recommended for use in age estimation of the younger individual (1,16,17).

All of the editions of the Greulich and Pyle atlas are based on data collected between 1931 and 1942 during the Brush Study (5,7,18). This study was a longitudinal study that collected serial anthropometric data and radiographs of children as they progressed through childhood and was developed by T. Wingate Todd as a method of tracking and measuring the development of children during this maturational process (19). Part of the criteria for inclusion in the study was a history of good health and normal development on the part of the child. The socioeconomic status of the children was defined by the authors as being “high.”

The 1959 edition of the atlas consists of two series of plates: One series follows female development, and the second follows the skeletal development of males (5). The process for development of the atlas followed a set formula. The team identified skeletal changes which they named “maturity indicators”; once these were identified, 100 radiographs were chosen that were most representative of that stage which were in turn arranged according to the listed maturity indicators. From this shortlist, the radiograph that most closely represented the identified phase of maturation was selected for inclusion in the atlas (5).

The aim of the current study was to determine the reliability of the Greulich and Pyle atlas when utilized as a method of age estimation for a modern Scottish sample to assess the validity and robusticity of this approach for the purposes of forensic age evaluation in the living.

## Methodology

Male and female left-hand/wrist radiographs were collected at Ninewells Hospital in Dundee, Scotland. Ninewells Hospital is a large teaching hospital which serves the local Tayside area in the East of Scotland. The population of the area consists of around 400,000 individuals, of which around 17% live in poverty as defined by the Scottish Indices of Multiple Deprivation, 20% are students who attend the local universities, and *c*. 1.9% are considered to be nonwhite. It should be noted that a large dependence on agriculture means that there is an increase in migrant workers on a seasonal basis. Life expectancy is 78.8 years (females 80.6 years, males 76.9 years), slightly higher than the national average (20).

Radiographs were collected from patients between the ages of birth and 21 years of age which had been taken when the patient had accessed the Accident and Emergency Department of the hospital. Ethical approval was granted by Ninewells Hospital for the collection of the anonymized images. Personal details were limited to sex, date of birth, date of image, and side of the body. Chronological age was calculated by the difference between date of birth and the date that the image was taken. A total of 406 left-hand/wrist radiographs (157 females and 249 males) were collected. Table 1 shows the numbers of images for each sex by age.

**Table:** Number of radiographic images separated by sex and age

Years	Female Left	Male Left	Total
1	3	3	6
2	3	3	6
3	3	3	6
4	6	6	12
5	0	7	7
6	8	2	10
7	7	8	15
8	3	8	11
9	11	12	23
10	19	15	34
11	6	17	23
12	11	15	26
13	17	16	33
14	10	18	28
15	5	21	26
16	10	19	29
17	7	21	28
18	12	19	31
19	6	19	25
20	10	17	27
	157	249	406

An estimation of age was undertaken for each of the radiographs using the Greulich and Pyle atlas (5). The age estimation was undertaken without prior knowledge of the chronological age of each of the children examined. Owing to well-recorded differences in the development of females and males, age estimation was undertaken separately for each sex (21–24).

Intra-observer accuracy was tested using a subset of 30 randomly selected radiographs from the female left-hand radiographs and 30 randomly selected radiographs from the male set of radiographs. These were observed 3 months after the first age estimation was undertaken. An inter-observer test was devised using 30 randomly selected female left-hand/wrist radiographs. The second assessor is a practicing forensic anthropologist with knowledge of, but not experience with, the Greulich and Pyle atlas. For the purposes of this test, the observer was given no additional instructions in the use of the atlas, was blind to the chronological age and was only informed of the sex of the individual.

## Results

Both the chronological ages and estimated ages were translated from years into months for the purposes of statistical analysis.

The 1959 edition of the Greulich and Pyle atlas has separate standards for males and females: In males, the image at which full skeletal maturity has been achieved is “*Male standard 31*,” which is assigned a chronological age of 19 years. For females, the corresponding image is that of “*Female Standard 27*,” which is assigned a chronological age of 18 years. In this study, all of the radiographs were age-estimated up to and including 20 years of age to confirm when age-related maturation could no longer be identified in the current sample. Within the 18- to 20-year age groups for females, there were 14 individuals who had not reached the stage of maturity seen in “*Female Standard 27*,” and in the 19- to 20-year age groups for males, there were 11 individuals who had not reached “*Male Standard 31*,” despite the individual having passed the identified chronological age for these standards. Finding individuals who were still undergoing fusion was not unexpected because in any population there will be individuals who, for a variety of reasons, achieve maturational milestones at a different chronological age to others (25,26). The radiographs in the Greulich and Pyle atlas represent the average or median skeletal development for that chronological age and do not illustrate outliers. Because these outliers were shown to exist in this cohort, all images were included in the statistical assessments as this is a true representation of the sample.

**Figure 1:**
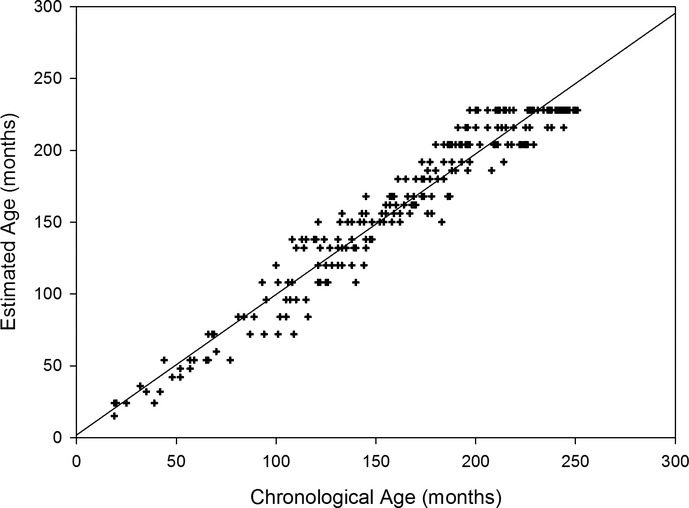
Linear regression between chronological age and estimated age using the Greulich and Pyle atlas for female left hand.

**Figure 2:**
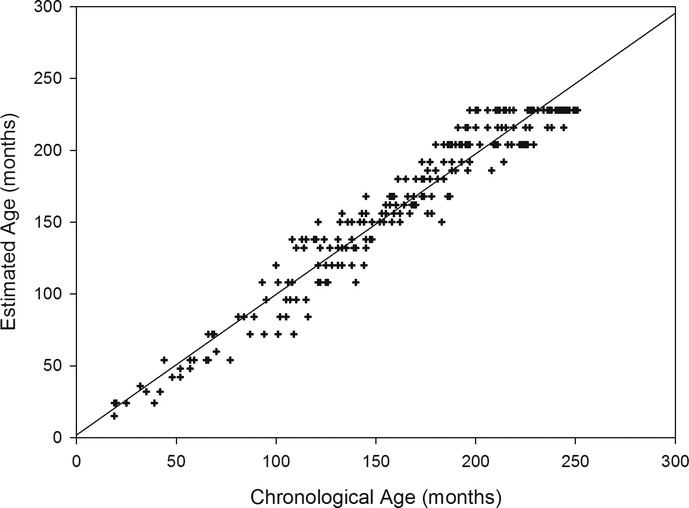
Linear regression between chronological age and estimated age using the Greulich and Pyle atlas for male left hand.

Linear regression analysis was undertaken on the data with estimated age treated as the independent variable in all of the calculations. Table 2 and Figs 1 and 2 present the results of this analysis. The *R*^2^ value for females is 0.939 and for males is 0.940; both of these values are highly significant (*p* < 0.001).

**Table:** *R*^2^ values and regression coefficients by sex for the age estimations compared to chronological age as undertaken by the first observer

	Regression Coefficient	*R*^2^ Value	*p*-Value
Female left hand/wrist	0.894	0.939	<0.001
Male left hand/wrist	0.979	0.940	<0.001

The relationship between chronological age and estimated age was tested for significance through a Mann–Whitney test. For both females and males, the difference between chronological age and estimated age using the Greulich and Pyle atlas was not significant (females *p* = 0.771, males *p* = 0.899).

**Figure 3:**
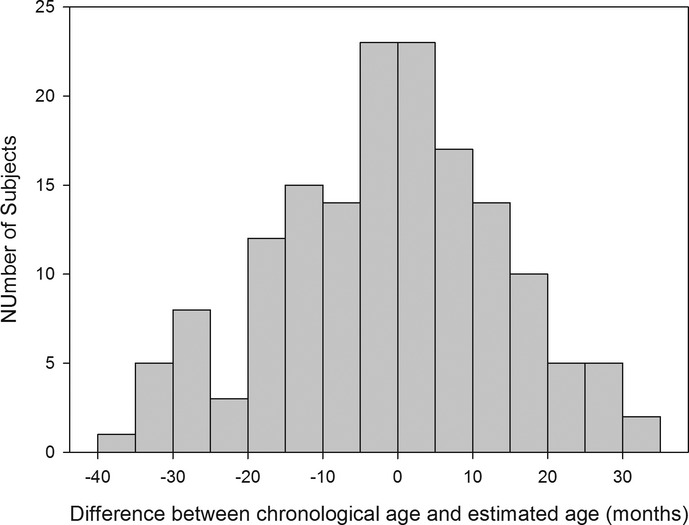
Mean differences between chronological age and estimated age (months) for female left-hand images.

**Figure 4:**
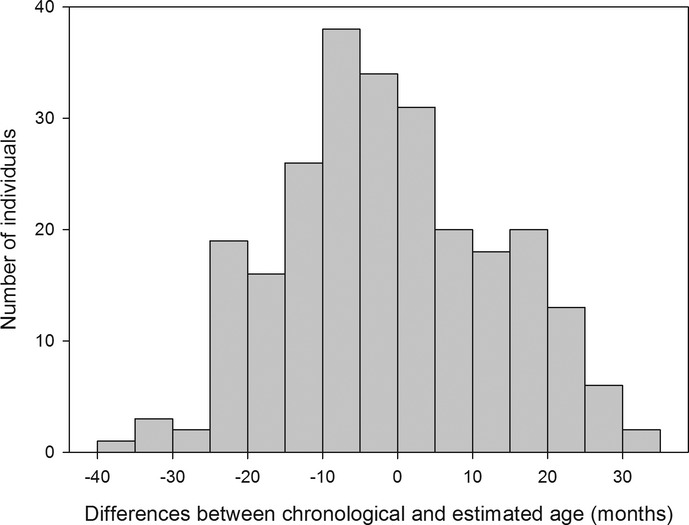
Mean differences between chronological age and estimated age (months) for male left-hand images.

The differences between the chronological ages and estimated ages were calculated by subtracting the chronological age from the estimated age. A negative value therefore indicates that the individual had been under-aged, and a positive value indicates an individual who had been over-aged using the Greulich and Pyle atlas (5). The differences between chronological age and age as estimated by the Greulich and Pyle atlas ranged from between an under-age of 37 months and an over-age of 31 months for both females and males; both sets of differences show a Gaussian distribution (Figs 3 and 4). The mean difference between chronological age and estimated age for each sex is negative in value (Table 3), indicating that on average, within this sample, the chronological age is in advance of the estimated age (−1.95 months for females and −1.63 months for males). The standard deviations for these groups, by sex, are 14.97 months for females and 14.16 months for males.

**Table:** Mean differences between chronological age and estimated age (months)

	Mean Difference Between Chronological Age and Estimated Age (Months)	Standard Deviation	Maximum Differences (Over- and Under-Age)
Female left hand/wrist	−1.95	14.97	Max 31.00
			Min −37.00
Male left hand/wrist	−1.63	14.16	Max 31.00
			Min −37.00

To obtain a more detailed picture of the differences between chronological age and estimated age, the data were broken down into age cohorts of 5 years (Table 4). It can be seen in Table 4 that for females, age is consistently over-estimated by between 2.04 and 3.06 months from 0 to 15 years of age. Table 4 also shows that for males, age is under-estimated from birth to 10 years of age by between 2.44 and 3.54 months and over-estimated by 1.74 months for 11- to 15-year-olds. The trend for both sexes in the 16- to 20-year age groups is a lag between estimated age and chronological age; this latter under-aging is to be expected because the atlas for both the male and female groups cannot assess age past the point at which maturity is achieved; although radiographs were collected and estimated up until the 20th year, there were only a small number of individuals who were still experiencing fusion at this time.

**Table:** Mean differences between estimated and chronological age in females and males by 5-year age cohorts

Age Cohort	Female Left Hand/Wrist Mean Difference by Cohort	Female Left Hand/Wrist Maximum and Minimum Differences	Female Left Hand/Wrist Standard Deviation	Male Left Hand/Wrist Mean Differences by Cohort	Male Left Hand/Wrist Maximum and Minimum Differences	Male Left Hand/Wrist Standard Deviation
0–5 years	2.25 (*n* = 16)	14.00 months	9.85	−3.54 (*n* = 22)	10 months	7.06
		−15.00 months			−15 months	
6–10 years	2.04 (*n* = 48)	31.00 months	13.36	−2.44 (*n* = 45)	30 months	17.25
		−28.00 months			−37 months	
11–15 years	3.06 (*n* = 50)	31.00 months	13.46	1.74 (*n* = 87)	25 months	12.95
		−33.00 months			−36 months	
16–20 years	−13.38 (*n* = 45)	23.00 months	14.05	−3.87 (*n* = 95)	31 months	14.42
		−37.00 months			−28 months	

The differences between chronological age and estimated age were broken down further into year cohorts for each sex (Table 5). The numbers of images in the younger groups are very small with larger numbers of individuals in older age groups. For the females, prior to the age of 9 years, there is a mixed pattern of under- and over-aging, although for the majority of groups for which there were data the trend was to over-age by between 1.14 and 5.12 months. From the age of 9, the atlas consistently over-ages females by between 0.20 and 5.73 months until the age of 17 years when the trend reverses because of the completion of the atlas series for females. For males, there is a tendency to over-age individuals between the ages of birth and 2 years of age; after this, the Greulich and Pyle atlas under-ages the majority of age groups by between 0.2 and 10 months, except for boys between the age of 9 and 10 who are over-aged by 2.92 months. The atlas consistently over-ages boys from the age of 13 years to 17 years of age by between 1.62 months and 11.05 months. At 18 years of age, this trend reverses again because of the completion of the atlas series.

**Table:** Differences between chronological and estimated age by age cohort in months

Age Cohort (years)	Female Left Hand/Wrist	Male Left Hand/Wrist
1	3 (*n* = 3)	1.67 (*n* = 3)
2	1.33 (*n* = 3)	0.0 (*n* = 3)
3	4.33 (*n* = 3)	−5.00 (*n* = 3)
4	−0.5 (*n* = 6)	−6.17 (*n* = 6)
5	–	−4.43 (*n* = 7)
6	5.12 (*n* = 8)	−10.0 (*n* = 2)
7	1.14 (*n* = 8)	−7.88 (*n* = 8)
8	−4.67 (*n* = 3)	−7.38 (*n* = 8)
9	5.73 (*n* = 11)	2.92 (*n* = 12)
10	0.00 (*n* = 19)	−0.2 (*n* = 15)
11	1.67 (*n* = 7)	−0.53 (*n* = 17)
12	5.09 (*n* = 11)	−0.94 (*n* = 15)
13	5.06 (*n* = 17)	1.62 (*n* = 16)
14	0.20 (*n* = 10)	0.00 (*n* = 18)
15	4.2 (*n* = 5)	7.09 (*n* = 21)
16	2.00 (*n* = 10)	11.05 (*n* = 19)
17	−7.86 (*n* = 7)	2.52 (*n* = 21)
18	−10.83 (*n* = 12)	−7.21 (*n* = 19)
19	−21.67 (*n* = 6)	−9.53 (*n* = 19)
20	−30.70 (*n* = 10)	−18.41 (*n* = 17)

The intra-observer tests involved retesting 30 randomly chosen images from the male left-hand images and 30 randomly chosen images from the female left-hand images. Regression analysis was undertaken on the results of the age intra-observer age estimations. The regression coefficients and *R*^2^ values are presented in Table 6. For the female intra-observer test, the *R*^2^ value = 0.973 and for the male intra-observer test, the *R*^2^ value = 0.963. A *t*-test of the intra-observer results indicate that there was no significant difference between the two sets of observations for either the female left-hand (*p* = 0.925) or the male left-hand images (*p* = 0.859).

**Table:** The results of the linear regression undertaken on the interobserver and intra-observer age estimations by sex

	Regression Coefficient	*R*^2^ Value	*p*-Value
Intra-observer results for female left hand/wrist	0.930	0.973	<0.001
Intra-observer results for male left hand/wrist	0.955	0.963	<0.001
Inter-observer results for female left hand/wrist	0.905	0.940	<0.001

The inter-observer test involved the age estimation of 30 randomly selected radiographs of female left hand/wrists. Linear regression was undertaken to examine the correlation between estimated age and chronological age for the age estimations undertaken by the second examiner, and the *R*^2^ value for this analysis was 0.940 (*p* < 0.001). The inter-observer results were compared to the analysis performed by the first observer using a *t*-test which indicated that there was no significant difference between the two sets of results (*p* = 0.982).

## Discussion

This project sought to test the Greulich and Pyle atlas method of age estimation on a modern Scottish population. In light of the recent Law Commission report (15) in England and Wales, the re-examination of anthropological methodologies is appropriate, especially those which are applied in ways for which they were never originally designed and which are highly likely to be presented to a court of law. The Greulich and Pyle atlas is one of these techniques; in addition to being applied in novel ways, it is also based on the development of children who were maturing in 1930’s America, creating a situation in which not only secular change but also differences in ethnicity and access to medical and nutritional resources could be widely altered in those who are undergoing age estimation to those whose images assisted in the creation of the atlas (27,28). An understanding of the reliability and validity of a method to the population that it is being applied is vital in these circumstances.

Owing to the ethical considerations of undertaking longitudinal radiographic studies on maturing children, it is not possible to develop modern equivalents of the radiographic atlases and so it has become necessary to test existing methods to understand inherent errors if the technique is applied to a targeted population. This study on a Scottish population resulted in good correlations between estimated age and chronological age by both observers, a finding that remained consistent for both males and females. Other studies have also found that the correlation between assessed age and chronological age is strong (29–41). However despite this, many authors argue that the Greulich and Pyle atlas either should be applied with population-specific modifications (29,31,33,36,38,40,42–47) or should be combined with other age estimation techniques for increased accuracy (35,48). There are also a number of studies which find that the Greulich and Pyle atlas is inappropriate for use on the population that they studied (49–52). These latter studies were arguably of populations in which access to nutrition and health care was reduced in comparison with a Western population such as is found in Scotland. These studies support the findings of Schmeling et al. who argued that both socioeconomic factors and ethnicity should be taken into consideration when undertaking a forensic age estimation (53).

The population studied here showed a pattern of under-estimating the age of males prior to puberty (13 years) and over-aging after puberty. This pattern for males is reflected in other studies (33,44,46,47,50,52,54). The pattern for females was different because, with the exception of two groups, the atlas tended to over-estimate age throughout the maturation process. Postpuberty, the atlas consistently over-aged females in the group, which is in agreement with the findings of other studies (29,31,33,36,47,52). These results indicate that the process of maturation which Greulich and Pyle aimed to illustrate has remained the same but it is the timings of the process which exhibit variation.

The mean of the difference between estimated age and chronological age ranges from 0 months (2-year-old males and 10-year-old females) to 11.05 months (16-year-old males). The maximum differences between chronological age and estimated age, however, showed a maximum under-age of 37 months (3.1 years) for both males and females and a maximum over-age by 31 months for both sexes. A difference between estimated age and chronological age of this magnitude means that in a forensic situation, the estimated age assigned through the use of the Greulich and Pyle atlas alone could result in a 3.1-year under-age or a 2.5-year over-age.

For younger individuals, the maximum under-age is 15 months for both females and males and the maximum over-age is 14 months for females and 10 months for males. This smaller range of over-aging and under-aging in the younger individual is in agreement with other studies which also found that the difference between age as estimated by the Greulich and Pyle atlas and chronological age is smaller in younger individuals (38,42,46,47). Care should be taken with the conclusions within this study as the numbers in the younger age groups are small.

In this study, the standard deviations across the male and female groups as a whole were 14.97 months for females and 14.16 months for males. When groups are broken down into 5-year cohorts, the standard deviation is noticeably smaller in the 0- to 5-year age groups for both sexes: 9.85 months for females and 7.06 months for males. The standard deviation was not calculated for the year cohorts owing to the small sample sizes in many of these groups. The Greulich and Pyle atlas presents two sets of tables containing standard deviations: The first set contain standard deviations derived from a test of the Todd standards (12) on the Brush data, and the second set are derived from a test of their own standard on data derived from a longitudinal study in Boston, MA (5). This study has provided standard deviations that are appropriate for use in age estimation undertaken on a child from a modern Scottish population, as suggested by Greulich and Pyle any age range should be given to two standard deviations, although the possibility of outliers must always be presented.

The level of agreement between inter- and intra-observer assessments in this study is high, agreeing with the findings in other studies where the reproducibility of the Greulich and Pyle atlas has been shown to be high (35,39,43,46,50,55). It is worth noting that while there is no significant difference between the first set of age estimations and the second set as undertaken by the first observer, there is a slight increase in the *R*^2^ value from the first to the second group females improved from *R*^2^ = 0.939 to *R*^2^ = 0.973 and males improved from *R*^2^ = 0.940 to *R*^2^ = 0.963, which may suggest that with experience the accuracy of age estimations increased for this practitioner. This agrees with the findings of Roche et al. (56) who found that intra-observer reliability did increase slightly with practice and experience, a finding supported by other authors who found slight differences in accuracy between experienced and nonexperienced assessors (37,45).

## Conclusion

Any modern test of an age estimation methodology that involves radiographs has to be undertaken using cross-sectional data because of the ethical considerations inherent in any imaging techniques that involve health risks. The radiographs used in this study were sourced from a local hospital and were screened only for major disorders, pathology and previous fractures. It was not possible to screen individuals for background, but it was felt that this is more representative of a forensic population where the background and history of the individual being age-estimated is rarely known in all but the most basic detail.

It is not possible for health reasons to recreate a longitudinal study of the type which provided the data which underpin the Greulich and Pyle atlas; therefore, it is necessary to understand the reliability and accuracy of this system of age estimation in living individuals. Studies such as this provide the forensic practitioner with increased data with which to support the age estimations that they undertake. The correlations found indicate that there is a strong relationship between estimated age and chronological age. This supports the argument that the use of this atlas is appropriate when estimating age in an individual from this modern population; however, the differences that have been found between chronological age and estimated age must be taken into consideration whenever this method is applied forensically. This study therefore supports the use of the Greulich and Pyle atlas when age estimating a child from this population as long as the differences highlighted in this study are taken into account.

## References

[1] Schmeling A, Grundmann C, Fuhrmann A, Kaatsch HJ, Knell B, Ramsthaler F (2008). Criteria for age estimation in living individuals. Int J Legal Med.

[2] De Roo T, Scröder HJ (1976). Pocket atlas of skeletal age.

[3] Gilsanz V, Ratib O (2005). Hand bone age.

[4] Gök Ş, Erölçer N, Özen C (1985). Age determination in forensic medicine, 2nd edn. Istanbul: Turkish Republic Ministry of Justice, Council of Forensic Medicine Press.

[5] Greulich WW, Pyle SI (1959). Radiographic atlas of skeletal development of the hand and wrist.

[6] Poland J (1898). Skiagraphic atlas showing the development of the bones of the wrist and hand.

[7] Pyle SI, Waterhouse AM, Greulich WW (1971). A radiographic standard of reference for the growing hand and wrist.

[8] Tanner JM, Healy MJR, Goldstein H, Cameron N (2001). Assessment of skeletal maturity and prediction of adult height (TW3 method).

[9] Tanner JM, Whitehouse RH, Healy MJR (1962). A new system for estimating skeletal maturity from the hand and wrist with standards derived from a study of 2600 healthy British children. Part II. The scoring system.

[10] Tanner JM, Whitehouse RH, Marshall WA, Healy MJR, Goldstein H (1975). Assessment of skeletal maturity and prediction of adult height.

[11] Thiemann H-H, Nitz I, Schmeling A (2006). Rontgenatlas der normalen hand im kindesalter.

[12] Todd TW (1937). Atlas of skeletal maturation.

[13] Beh P, Payne-James J, Black SM, Aggrawal A, Payne-James J (2010). Clinical and legal requirements for age determination in the living. Age estimation in the living.

[14] Crawley H (2007). When is a child not a child?.

[15] The Law Commission (2011). Expert evidence in criminal proceedings in England and Wales.

[16] Schmeling A, Olze A, Reisinger W, Geserick G (2001). Age estimation of living people undergoing criminal proceedings. Lancet.

[17] Schmeling A, Olze A, Reisinger W, Rosing FW, Geserick G (2003). Forensic age diagnostics of living individuals in criminal proceedings. HOMO.

[18] Greulich WW, Pyle SI (1950). Radiographic atlas of skeletal development of the hand and wrist.

[19] Keith A (1939). In memoriam. Thomas Wingate Todd (1885–1938). J Anat.

[20] General Register Office for Scotland (2009). Directgov. Angus council area-demographic factsheet.

[21] Loesch DZ, Hopper JL, Rogucka E, Huggins RM (1995). Timing and genetic rapport between growth in skeletal maturity and height around puberty: similarities and differences between girls and boys. Am J Hum Genet.

[22] Pryor JW (1923). Differences in the time of development of centres of ossification in the male and female skeleton. Anat Rec.

[23] Pryor JW (1925). Time of ossification of the bones of the hand of the male and female. Am J Phys Anthropol.

[24] MacKay D (1952). Skeletal maturation in the hand: a study of development in East African children. Trans R Soc Trop Med Hyg.

[25] Hagg U, Taranger J (1992). Pubertal growth and maturity pattern in early and late maturers. A prospective longitudinal study of Swedish urban children. Swed Dent J.

[26] Lopez-Blanco M, Izaguirre-Espinoza I, Macias-Tomei C, Saab-Verardy L (1995). Growth in stature in early, average, and late maturing children of the Caracas mixed-longitudinal study. Am J Hum Biol.

[27] Schmeling A, Reisinger W, Loreck D, Vendura K, Markus W, Geserick G (2000). Effects of ethnicity on skeletal maturation: consequences for forensic age estimations. Int J Legal Med.

[28] Schmeling A, Schulz R, Danner B, Rosing FW (2006). The impact of economic progress and modernization in medicine on the ossification of hand and wrist. Int J Legal Med.

[29] Büken B, Safak AA, Yazici B, Büken E, Mayda AS (2007). Is the assessment of bone age by the Greulich-Pyle method reliable at forensic age estimation for Turkish children?. Forensic Sci Int.

[30] Bull RK, Edwards PD, Kemp PM, Fry S, Hughes IA (1999). Bone age assessment: a large scale comparison of the Greulich and Pyle, and Tanner and Whitehouse (TW2) methods. Arch Dis Child.

[31] Calfee R, Sutter M, Steffen J, Goldfarb C (2010). Skeletal and chronological ages in American adolescents: current findings in skeletal maturation. J Child Orthop.

[32] Chan ST, Chang KSF, Hsu FK (1961). Growth and skeletal maturation of Chinese children in Hong Kong. Am J Phys Anthropol.

[33] Chiang K-H, Chou AS-B, Yen P-S, Ling C-M, Lin C-C, Lee C-C (2005). The reliability of using Greulich-Pyle method to determine children’s bone age in Taiwan. Tzu Chi Med J.

[34] Cole AJL, Webb L, Cole TJ (1988). Bone age estimation: a comparison of methods. Br J Radiol.

[35] Garamendi PM, Landa MI, Ballesteros J, Solano MA (2005). Reliability of the methods applied to assess age minority in living subjects around 18 years old. A survey on a Moroccan origin population. Forensic Sci Int.

[36] Griffith JF, Cheng JCY, Wong E (2007). Are western skeletal age standards applicable to the Hong Kong Chinese population? A comparison of the Greulich and Pyle method and the Tanner and Whitehouse method. Hong Kong Med J.

[37] Groell R, Lindbichler F, Riepl T, Gherra L, Roposch A, Fotter R (1999). The reliability of bone age determination in central European children using the Greulich and Pyle method. Br J Radiol.

[38] Mora S, Boechat M, Pietka E, Huang HK, Gilsanz V (2001). Skeletal age determinations in children of European and African descent: applicability of the Greulich and Pyle standards. Pediatr Res.

[39] Van Rijn RR, Lequin MH, Robben SGF, Hop WCJ, Van Kuijk C (2001). Is the Greulich and Pyle atlas still valid for Dutch Caucasian children today?. Pediatr Radiol.

[40] Zhang A, Sayre JW, Vachon L, Liu BJ, Huang HK (2009). Racial differences in growth patterns of children assessed on the basis of bone age. Radiology.

[41] Berst MJ, Dolan L, Bogdanowicz MM, Stevens MA, Chow S, Brandser EA (2001). Effect of knowledge of chronological age on the variability of pediatric bone age determined using the Greulich and Pyle standards. Am J Roentgenol.

[42] Loder R, Estle DT, Morrison K, Eggleston D, Fish D, Greenfield M (1993). Applicability of the Greulich and Pyle skeletal age standards to black and white children today. Am J Dis Child.

[43] Tisè M, Mazzarini L, Fabrizzi G, Ferrante L, Giorgetti R, Tagliabracci A (2011). Applicability of Greulich and Pyle method for age assessment in forensic practice on an Italian sample. Int J Legal Med.

[44] Koc A, Karaoglanoglu M, Erdogan M, Kosecik M, Cesur Y (2001). Assessment of bone ages: is the Greulich-Pyle method sufficient for Turkish boys?. Pediatr Int.

[45] Koski K, Haataja J, Lappaleinen M (1961). Skeletal development of the hand and wrist in Finnish children. Am J Phys Anthropol.

[46] Ontell FK, Ivanovic M, Ablin DS, Barlow TW (1996). Bone age in children of diverse ethnicity. Am J Roentgenol.

[47] Rikhasor RM, Qureshi AM, Rathi SL, Channa NA (1999). Skeletal maturity in Pakistani children. J Anat.

[48] Schmidt S, Koch B, Schulz R, Reisinger W, Schmeling A (2008). Studies in the use of the Greulich-Pyle skeletal age method to assess criminal liability. Leg Med.

[49] Patil ST, Parchand MP, Meshram MM, Kamdi NY (2012). Applicability of Greulich and Pyle skeletal age standards to Indian children. Forensic Sci Int.

[50] Zafar AM, Nadeem N, Husen Y, Ahmad MN (2010). An appraisal of Greulich-Pyle Atlas for skeletal age assessment in Pakistan. J Pak Med Assoc.

[51] Lewis CP, Lavy CBD, Harrison WJ (2002). Delay in skeletal maturity in Malawian children. J Bone Joint Surg Am.

[52] Nahid G, Abdorrahim A, Gharib SM, Anvar E (2010). Assessment of bone age in Kurdish children in Iran. Pak J Med Sci.

[53] Schmeling A, Olze A, Reisinger W, Geserick G (2005). Forensic age estimation and ethnicity. Leg Med.

[54] Büken B, Erzengin OU, Büken E, Safak AA, Yazici B, Erkol Z (2009). Comparison of the three age estimation methods: which is more reliable for Turkish children?. Forensic Sci Int.

[55] Lynnerup N, Belard E, Buch-Olsen K, Sejrsen B, Damgaard-Pedersen K (2008). Intra- and interobserver error of the Greulich-Pyle method as used in a Danish forensic sample. Forensic Sci Int.

[56] Roche AF, Rohmann CG, French NY, Davila GH (1970). Effect of training on replicability of assessments of skeletal maturity (Greulich-Pyle). Am J Roentgenol.

